# Latent profiles of parental attachment styles and their associations with parenting behaviors among parents of school-aged children

**DOI:** 10.3389/fpsyg.2026.1691655

**Published:** 2026-05-04

**Authors:** Yali Wang, Xiaoshu Lin, Xinxin Zhang, Xiaoyun Ma, Ciqing Bao, Xiaofan Chen

**Affiliations:** 1 Department of Psychotherapy, Wenzhou Seventh People’s Hospital, Wenzhou, China; 2 Shatou Town Central Primary School, Wenzhou, Zhejiang, China

**Keywords:** attachment anxiety, attachment avoidance, interpersonal acceptance rejection theory, Latent Profile Analysis, parenting behavior

## Abstract

This study utilized Latent Profile Analysis (LPA) to identify attachment style latent profiles among parents of school-aged children and examine their association with parenting behaviors. Analysis of attachment anxiety and avoidance dimensions revealed four distinct parental profiles: low anxiety/avoidance, high anxiety/moderate avoidance, moderate anxiety/avoidance, and high anxiety/avoidance. Low anxiety/avoidance parents demonstrated significantly greater acceptance than all other latent profiles. Conversely, parents in high anxiety profiles exhibited significantly more rejecting behaviors than low anxiety latent profile. Notably, no sex differences emerged in profile distribution or parenting patterns. These findings reveal heterogeneous parental attachment configurations and establish attachment style as a robust correlate of parenting. This highlights the critical link between parental attachment representations and caregiving practices.

## 1 Introduction

Attachment is a lasting, intense, and emotionally close bond formed between an individual and their significant others. This bond has a profound impact on the development of an individual’s emotional, cognitive, and social behaviors (Slade and Holmes, 2019). Parents are children’s primary attachment figures, and children’s sense of security, as well as their emotional and psychological states, depend on the quality of their relationship with their parents (Jones et al., 2014; Ravitz et al., 2010). Individuals with a secure attachment style typically possess stronger emotional awareness and empathy, which makes them more likely to adopt warm and sensitive parenting behaviors. In contrast, parents with anxious or avoidant attachment styles may exhibit inconsistent or distant parenting behaviors due to deficits in emotional regulation (Gross et al., 2023; Jones et al., 2015). Beyond indirectly regulating parenting behaviors through emotions, parental attachment can directly influence parenting behaviors (Deneault et al., 2021; Li et al., 2021; Wang et al., 2024). Attachment avoidance and anxiety positively predict negative parenting behaviors, impairing the parent and child attachment bond and increasing the risk of behavioral problems among their children (Li et al., 2021; Matetovici et al., 2025). Therefore, an in-depth exploration of the inherent connections between parental attachment patterns and parenting behaviors will provide a key basis for scientifically guiding parent and child interaction strategies and enhancing parenting competence.

The Interpersonal Acceptance Rejection Theory (IPAR Theory) is an evidence-based framework that aims to analyze the dynamic mechanisms of how acceptance and rejection influence individuals from a lifespan development perspective (Rohner and Lansford, 2017; Rohner, 1986). This theory asserts that humans require positive responses from significant others particularly parents and intimate partners throughout development (Rohner and Lansford, 2017). Childhood acceptance constitutes love, care, attention, and support from caregivers, whereas interpersonal rejection typically manifests as marked coldness, affectional neglect, hostility, or indifference (Rohner et al., 2012). Empirical evidence confirms that warm, affirmative parenting behaviors enhance children’s psychological resilience while mitigating the detrimental impacts of caregiver rejection on emotional, cognitive, and behavioral functioning (Meng et al., 2024; Morgan et al., 2020). Although moderate rejection may foster psychological defense mechanisms and autonomy, rejection exceeding individual tolerance thresholds elevates risks of internalizing symptoms (primarily anxiety, depression, and social withdrawal) and externalizing symptoms (notably rule breaking and aggression) (Folker et al., 2024; Rohner and Lansford, 2017).

A profound connection exists between attachment patterns and parenting behaviors. Based on observations of infant and caregiver dyads, Bowlby (1982) proposed that infants’ interactive experiences with primary caregivers form an “internal working model.” This model functions as a cognitive framework guiding self-other understanding, with its internalized attachment strategies potentially persisting across generations through parenting behavior transmission allowing attachment patterns to reemerge in parent and child interactions (De Carli et al., 2015; Jones et al., 2015). Although previous studies have empirically confirmed that parental attachment patterns exert multifaceted influences on parenting (Jones et al., 2015; Li et al., 2021; Wang et al., 2024), controversial or conflicting findings remain regarding the relationship between parental attachment styles and parenting behaviors. On one hand, most research has focused on the associations between negative attachment patterns (e.g., avoidant, anxious) and disruptive parenting behaviors (e.g., harsh punishment, emotional rejection) (Li et al., 2021; Nordahl et al., 2020; Wang et al., 2024), yet relatively few have explored the specific links between parents’ secure attachment tendencies and positive parenting strategies. On the other hand, research findings are divided on whether gender differences exist in the relationship between parental attachment styles and parenting behaviors. Li et al. (2021) study on parent and adolescent relationships found that fathers’ avoidant attachment has a stronger predictive effect on harsh parenting behaviors compared to mothers’, and this mechanism can weaken the quality of parent–child attachment. Wang et al. (2024) research revealed that mothers exhibit significantly lower levels of attachment anxiety and avoidance than fathers, alongside more positive parenting behaviors. A synthesis of recent studies indicates that, regardless of parental gender, insecure attachment in either parent compared to secure attachment significantly impacts relationships between parents and children (Dagan et al., 2021; Deneault et al., 2021). However, existing research also suggests that the relationship between attachment patterns and parenting behaviors may not be linear and may be influenced by multiple contextual factors, such as child temperament and cultural background (Brock and Kochanska, 2019; Williams et al., 2024). Against this complex backdrop, exploring the links between attachment patterns and parenting behaviors can deepen our understanding of the intergenerational transmission mechanisms of attachment patterns and parent and child interaction styles. This exploration also provides a theoretical basis for enhancing parenting competence and improving relationships between parents and children.

Additionally, previous research has predominantly classified parental attachment types based on theoretical assumptions or observed variables (Jones et al., 2015; Ravitz et al., 2010). Such *a priori* categorization may overlook potential heterogeneity within parental populations. Fine grained analyses employing multidimensional variables could identify attachment based parental subgroups to examine differential subgroup preferences in parenting behavior selection. Latent Profile Analysis (LPA)—a person centered, data driven methodology—can identify homogeneous population profiles using sets of observed indicators (Ferguson et al., 2020). This is the core rationale for choosing LPA in our study: it directly addresses the limitation of *a priori* categorization by capturing the fine grained, unobserved heterogeneity in parental attachment that traditional classification methods miss. LPA is performed based on the continuous dimensions of attachment anxiety and avoidance, serving as a complementary approach to the traditional dimensional perspective rather than negating the continuous nature of attachment (Wen et al., 2023). Several existing studies have applied LPA to explore how parent attachment subtypes influence parenting behaviors, focusing on adolescent developmental trajectories (Li et al., 2023; Russotti et al., 2023). However, while school-aged children’s psychological wellbeing demonstrates stronger parental attachment dependence than adolescents, no research has employed person centered methods to directly examine attachment parenting behavior linkages within this age cohort. Accordingly, focusing on the link between the attachment patterns and parenting behaviors of children’s parents allows us to investigate the critical role of attachment parenting dynamics in a cohort with heightened attachment needs, while also addressing the relative scarcity of person-centered research specific to this developmentally distinct stage.

Building upon this foundation, the present study applies Latent Profile Analysis (LPA) to examine differential linkages between parental attachment profiles and parenting behaviors among parents of children. This study aims to extend the literature in two key ways: first, it applies a person-centered approach to the understudied population of parents of school-aged children (a group with stronger attachment dependence than adolescents); second, it provides data driven, empirically derived parental attachment profiles that can serve as a framework for subsequent studies on attachment parenting behavior dynamics. This research pursues two primary objectives:

Objective 1: Identify the latent profile types of attachment among parents of school-aged children. Based on attachment theory, we hypothesize that there are different attachment profiles, such as combinations of characteristics like low anxiety/low avoidance (secure type), high anxiety, and high avoidance.

Objective 2: We aim to examine the relationship between different latent attachment profiles and parenting behaviors and explore sex differences and differences in parenting behaviors across different attachment profiles.

Based on previous research, we anticipate that parenting behaviors will be more strongly associated with attachment profiles than with sex. We hypothesize that there are significant differences in parenting behaviors across different attachment profiles; specifically, parents with low avoidance/low anxiety characteristics (secure type) are more likely to exhibit accepting than rejecting parenting behaviors.

## 2 Materials and methods

This study employed a cross-sectional design and utilized Latent Profile Analysis (LPA) to examine the associations between parental attachment profiles and parenting behaviors.

### 2.1 Participants

Participants were recruited via cluster sampling combined with convenience sampling: Five classes were randomly selected from all classes at Shatou Town Central Primary School in Wenzhou City, and all parents of students in these classes were invited to participate.

Inclusion criteria: Parents of school-aged children who were 6–12 years old, aged 22–60 years, proficient in Chinese, with at least a junior high school education, and who volunteered to take part in the study.

Exclusion criteria: Aged <22 or >60 years, history of psychotropic medication use/mental illness, or >10% missing questionnaire data.

In this survey, a total of 426 parents of primary school children initially participated, but 10 questionnaires were eliminated from the analysis because of the overly uniform responses provided. Ultimately, 416 valid questionnaires were retained for further examination, resulting in an effective rate of 97.65%.

### 2.2 Procedures

Survey data were collected using the Questionnaire Star software program, a tool widely utilized in survey research across China. The questionnaire link was distributed by the school teacher via the class WeChat group. After obtaining informed consent, participants completed a series of self-report measures. Funds were allocated to the classes of the participants’ children for the development of a class book corner. All aspects of the study, including the consent form, were approved by the Hospital Ethics Committee.

### 2.3 Instruments

#### 2.3.1 The Revised Adult Attachment Scale (RAAS)

The AAS was employed to assess adult attachment patterns. Originally developed by Collins and Read (1990) and revised by Collins (1996), this self-report measure comprises three subscales (closeness, dependence, anxiety) with 18 items rated on a five-point Likert scale. This scale was culturally adapted for Chinese populations by Wu et al. (2004). The closeness and dependence subscales assess an individual’s capacity for intimacy in close relationships, and reverse scoring of these subscales has been widely validated as a measure of attachment avoidance in prior research (Katznelson et al., 2025; Wei et al., 2024). To align with the classic two-dimensional framework of adult attachment, we combined and reverse scored these two subscales to derive the attachment avoidance dimension in the present study. Participants were classified into secure, anxious, dismissing, or fearful attachment types based on mean avoidance and anxiety scores. Participants with scores near the subscale midpoint of three were designated as having unclassified attachment, indicating that they did not meet the prototype thresholds. The scale showed high reliability in this study (Cronbach’s α = 0.760).

#### 2.3.2 Parental Acceptance/Rejection Questionnaire (PARQ)

The PARQ (Rohner and Khaleque, 2005) developed by Rohner was adopted as the research instrument to examine parental acceptance and rejection behaviors toward children. This study utilized the abbreviated version consisting of 24 items, organized into two primary dimensions. The parental acceptance dimension is measured by the warmth/acceptance factor. The parental rejection dimension comprises three sub-factors: hostility/aggression, indifference/neglect, and undifferentiated rejection. Responses were recorded on a four-point Likert scale ranging from 1 = “Almost never true” to 4 = “Almost always true.” Following reverse scoring of designated items, the mean score of the eight items in the acceptance dimension was computed as the parental acceptance score, with higher scores indicating greater perceived parental acceptance. The mean score of the 16 items constituting the parental rejection dimension was computed as the parental rejection score, with higher scores indicating greater perceived parental rejection. The questionnaire has demonstrated good reliability and validity, as evidenced by previous research conducted within Chinese cultural contexts (Putnick et al., 2012). In the current study, scale reliability was assessed, yielding a Cronbach’s α coefficient of 0.89, which indicates good internal consistency.

### 2.4 Data analysis

Statistical analyses were conducted using SPSS 27.0 and Mplus 8.7. Preliminary analyses employed SPSS to compute descriptive statistics and Pearson correlations for all variables.

Latent Profile Analysis in Mplus was used to determine the number and nature of parental attachment categories, based on responses to the 18-item Adult Attachment Scale (AAS). The AAS scores (ranging from 1 to 5) were treated as continuous variables, with scores on the two dimensions of avoidance and anxiety serving as classification criteria. To identify the optimal profile type, model fit indices were compared across five models with profiles ranging from 1 to 5. The selection of the optimal number of latent classes was based on multiple posterior fit statistics: Akaike Information Criterion (AIC), Bayesian Information Criterion (BIC), Adjusted BIC (aBIC), Lo-Mendell-Rubin Adjusted Likelihood Ratio Test (LMRT), Bootstrap Likelihood Ratio Test (BLRT), and entropy measures. The AIC, BIC (and sample size-adjusted BIC), and entropy were used to assess model quality. Lower values of AIC, BIC, and aBIC indicate better model fit, while entropy values closer to 1.0 indicate higher classification quality (Ferguson et al., 2020; Geiser, 2012). The LMRT and BLRT compare the fit of nested latent class models, specifically for models with an increasing number of classes; non-significant values (*p* > 0.05) suggest that the model with fewer classes should be accepted (Nylund-Gibson and Choi, 2018). Solutions with small classes (<5% of the total sample) were also considered because small groups can represent meaningful exceptions that are of substantive interest in this study (Geiser, 2012). To avoid local optima, latent profile estimation used 500 random starting values, 50 iterations, and retained 50 solutions for optimization. Bootstrap resampling (1,000 replications) validated the stability of small clusters and classification accuracy. Additionally, in this study, sex and age were included as covariates in the latent profile analysis to examine their effects on attachment types.

After determining the optimal number of profiles, the method proposed by Bolck, Croons, and Hagenaars (BCH) (Bolck et al., 2004) was used to test for differences in sex and parenting behaviors across different classes. The BCH method accounts for classification uncertainty, enabling robust comparisons between profiles.

## 3 Results

### 3.1 Preliminary analysis

Descriptive statistics for participants were presented in Table 1. Correlation analyses revealed distinct associations between attachment styles and parenting behaviors (Table 2). Rejection and its subfactors demonstrated significant positive correlations with both attachment avoidance and anxiety. Conversely, acceptance showed significant negative correlations with avoidance and anxiety.

**TABLE 1:** Demographic details of participants.

Role	Age (years) *M* ± SD	Education (years) *M* ± SD	Marital status, *n* (%)					Total
			Married	Divorced	Widowed	Separated	Unmarried	
Father	39.48 ± 4.98	11.87 ± 2.95	101 (95.28%)	4 (3.77%)	–	–	1 (0.94%)	106
Mother	37.90 ± 4.64	11.53 ± 3.13	297 (95.81%)	7 (2.26%)	2 (0.65%)	1 (0.32%)	3 (0.97%)	310

M, mean; SD, standard deviation.

**TABLE 2:** Correlation between attachment styles and parenting behaviors.

Variables	*M*	SD	1	2	3	4	5	6	7
1. Avoidance	2.690	0.440	1	–	–	–	–	–	–
2. Anxiety	2.040	0.790	0.456***	1	–	–	–	–	–
3. Acceptance	3.140	0.500	−0.207***	−0.253***	1	–	–	–	–
4. Rejection	1.530	0.490	0.233***	0.398***	−0.360***	1	–	–	–
5. Hostility	1.588	0.583	0.176***	0.353***	−0.276***	0.919***	1	–	–
6. Coldness	1.545	0.489	0.263***	0.417***	−0.389***	0.884***	0.670***	1	–
7. Undifferentiated rejection	1.402	0.550	0.196***	0.295***	−0.321***	0.906***	0.775***	0.735***	1

M, mean; SD, standard deviation. All correlations were significant at ***p < 0.001.

### 3.2 Latent profiles for attachment anxiety and avoidance

The LPA with attachment avoidance and anxiety dimensions as classification indicators showed that the four-profile model had the optimal fit. As shown in Table 3, with the increase in the number of profiles, the decreasing AIC/BIC values, converging log-likelihood estimates, and improving entropy values indicated enhanced model fit. When the number of profiles was 4, the BIC and aBIC values were the lowest. In addition, LMRT and BLRT for the five-profile solution did not reach a significant level (*p* > 0.05), indicating that the four-profile solution was better. The entropy index also supported the four-profile solution (>0.80), suggesting that this model could classify individuals into different categories with high accuracy. After including sex and age as covariates, the model fit indices (AIC = 1323.198, BIC = 1375.597) showed no significant difference from the baseline model without covariates (all *p* > 0.05), indicating that sex and age did not significantly affect the profile classification.

**TABLE 3:** Latent Profile Analysis model fit summary for attachment.

Profile	AIC	BIC	aBIC	Log likelihood	Entropy	pLMRT	pBLRT	Proportion for each profile (%)
1	1492.793	1508.916	1496.223	−742.396	–	–	–	100%
2	1394.391	1422.606	1400.393	−690.196	0.642	<0.001	<0.001	61.9% vs. 38.1%
3	1362.688	1402.995	1371.262	−671.344	0.757	0.001	<0.001	44.2% vs. 5.7% vs. 50%
4	1323.198	1375.597	1334.344	−648.599	0.85	0.005	<0.001	37.9% vs. 22.3% vs. 36.3% vs. 3.5%
5	1322.514	1387.005	1336.233	−645.257	0.874	0.077	0.364	36.1% vs. 37.6% vs. 22.1% vs. 3.9% vs. 0.3%

AIC, Akaike Information Criterion; BIC, Bayesian Information Criterion; aBIC, Adjusted BIC; *p*LMRT, Lo-Mendell-Rubin test *p*-value; *p*BLRT, Bootstrapped Likelihood ratio test *p*-value.

Notably, among the four latent profiles, there existed a small profile comprising less than 5% of the total sample size. Bootstrapping validation with 1,000 replications confirmed the solution’s robustness, evidenced by non-overlapping 95% confidence intervals across profile parameters and universally significant between profile differences (all *p* < 0.001). Membership consistency analysis revealed high classification stability, with 75.3% of participants maintaining identical profile assignments across iterations (posterior probability > 0.85).

**FIGURE 1:**
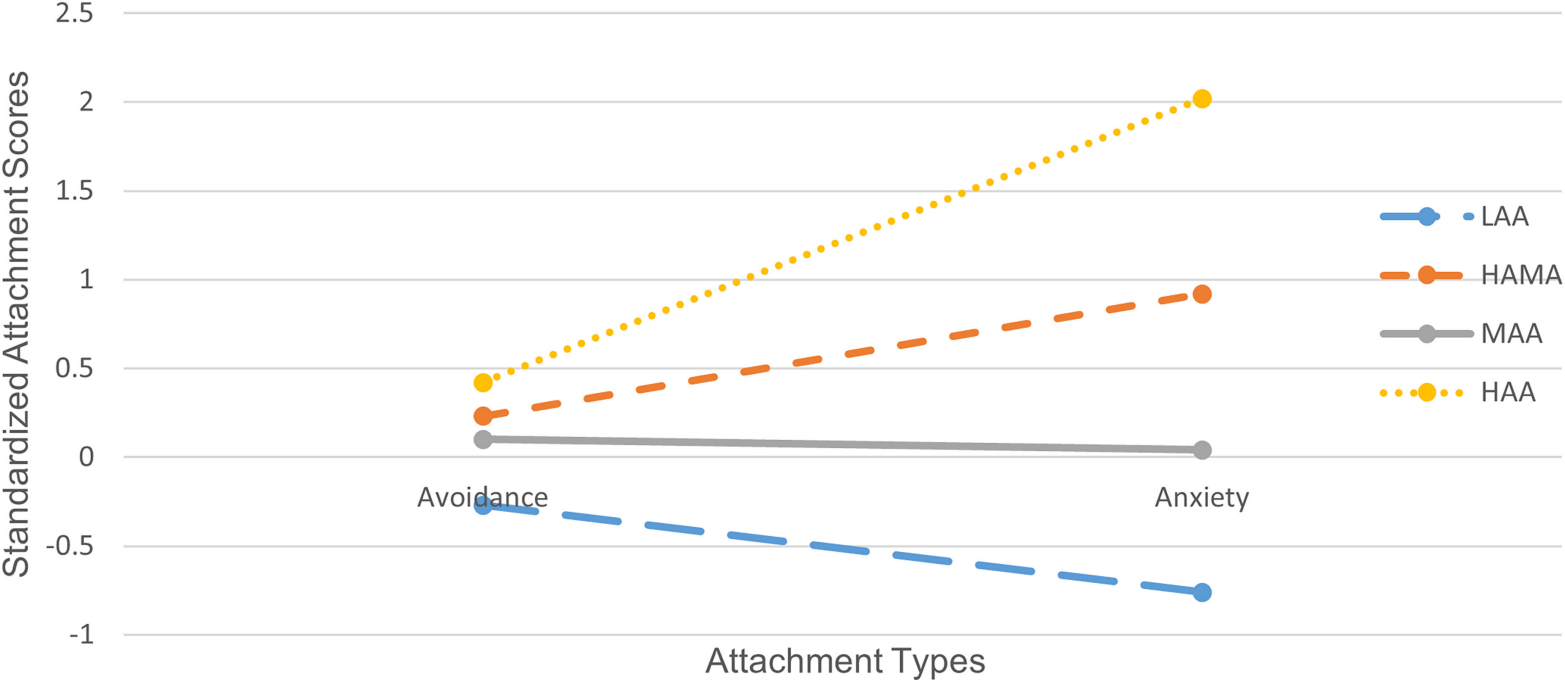
Latent profiles of attachment. LAA, low anxiety/avoidance; HAMA, high anxiety and moderate avoidance; MAA, moderate anxiety/avoidance; HAA, high anxiety/avoidance.

Figure 1 showed the estimates of the four latent profiles of attachment. The first latent profile, consisting of 158 respondents (37.9%), was characterized by the lowest estimates, which we named “Low Anxiety/Avoidance (LAA).” The second latent profile, comprising 93 respondents (22.3%), is marked by high anxiety estimates, and we named this profile “High Anxiety and Moderate Avoidance (HAMA).” The third latent profile, including 151 respondents (36.3%), features moderate estimates, so we named it “Moderate Anxiety/Avoidance (MAA).” The fourth latent profile, with 14 respondents (3.5%), was characterized by the highest estimates, and we named this profile “High Anxiety/Avoidance (HAA).”

### 3.3 Comparisons of parenting behaviors across latent profiles

The BCH procedure was used to examine differences in acceptance/rejection among the four latent profiles of attachment (Table 4). Bonferroni correction was applied to adjust the significance level (adjusted α = 0.05/6 ≈ 0.0083). The results indicated that LAA demonstrated significantly higher levels of acceptance than other latent profiles (χ^2^ = 25.44, *df* = 3, *p* < 0.0083). Compared to the latent profiles with moderate and low anxiety characteristics, the two latent profiles with high anxiety characteristics (HAMA, HAA) exhibited higher levels of rejection (χ^2^ = 68.013, *df* = 3, *p* < 0.0083). Specifically, HAMA scored significantly higher than MAA in rejection (χ^2^ = 7.511, *df* = 3, *p* < 0.0083), and HAA scored significantly higher than MAA in coldness (χ^2^ = 6.998, *df* = 3, *p* < 0.0083). HAMA exhibited significantly higher undifferentiated rejection compared to LAA (χ^2^ = 28.233, *df* = 3, *p* < 0.0083) and MAA (χ^2^ = 8.062, *df* = 3, *p* < 0.0083). Despite differing in avoidance, the two high anxiety profiles (HAMA/HAA) showed no significant differences in parenting behaviors (for acceptance: χ^2^ = 1.966, *df* = 3, *p* = 0.161; for rejection: χ^2^ = 0.473, *df* = 3, *p* = 0.492).

**TABLE 4:** Equality tests of means across attachment profiles for acceptance/rejection (BCH).

Variables	LAA	HAMA	MAA	HAA	Chi-square	Significant differences
Acceptance	3.299	3.02	3.09	2.723	25.44	LAA > HAMA, MAA, HAA
Rejection	1.304	1.779	1.567	1.895	68.01	HAA > MAA, HAMA > MAA > LAA
Hostility	8.139	11.168	9.779	11.673	51.339	HAMA, MAA, HAA > LAA
Coldness	7.803	10.671	9.681	12.008	81.823	HAMA > LAA, HAA > MAA > LAA
Undifferentiated rejection	4.926	6.628	5.609	6.644	33.523	HAMA > LAA, MAA

BCH, Bolck-Croon-Hagenaars method; LAA, low anxiety/avoidance; HAMA = high anxiety and moderate avoidance; MAA, moderate anxiety/avoidance; HAA, high anxiety/avoidance; Bonferroni correction was applied to all significance tests (adjusted α = 0.05/6 ≈ 0.0083).

## 4 Discussion

This study revealed the latent profiles of attachment styles among parents of school-aged children and their associations with parenting patterns through LPA, providing a new perspective for understanding the intergenerational transmission mechanism of attachment. The results indicate that there are stable classifications of attachment styles within the parental population, and parents with different attachment styles exhibit significant differences in their adopted parenting behaviors. The specific discussion is as follows:

### 4.1 Latent profiles of parental attachment styles

This study identified four attachment profiles through data driven LPA based on the dual-dimensional framework of attachment anxiety and avoidance: low anxiety/avoidance, high anxiety/moderate avoidance, moderate anxiety/avoidance, and high anxiety/avoidance. This outcome aligns substantially with Bartholomew and Horowitz’s four category model (secure, preoccupied, dismissing, fearful) (Bartholomew and Horowitz, 1991). Traditional studies have mostly classified attachment styles based on thresholds derived from theoretical measurements (Ravitz et al., 2010). This *a priori* grouping approach may overlook heterogeneous differences along continuous dimensions (Fraley and Roisman, 2019)—for instance, simplistically categorizing the transitional state of moderate anxiety/avoidance as “unclassified” or merging it into other categories. As a data driven method, LPA identifies natural clusters based on actual response patterns, allowing it to capture intermediate subgroups (such as moderate anxiety/avoidance) that are neglected in traditional classifications, thereby refining subgroup differences not fully captured by conventional categorization (Ferguson et al., 2020; Geiser, 2012).

Consistent with previous LPA studies on attachment styles (Shevlin et al., 2014; Vaillancourt-Morel et al., 2022), in the present study, the profile characterized by low anxiety and low avoidance (secure type) accounted for the largest proportion. Meanwhile, this study identified a small cluster (3.5%) among the parental population characterized by high anxiety and high avoidance, and the stability of this small cluster was validated through Bootstrap resampling. A synthesis of previous studies reveals that there are differences in size among attachment types (Ravitz et al., 2010). Vaillancourt-Morel et al. (2022) using LPA to study attachment styles in community residents, found that the profile of high attachment avoidance and low attachment anxiety accounted for only 7.6%, which was far smaller than the other three profile types. Shevlin et al.’s (2014) study on parents who had experienced the loss of a child showed that the proportions of the low anxiety/high avoidance profile (4.3%) and high anxiety/high avoidance profile (6.7%) were significantly lower than those of the other two attachment profiles (low avoidance/anxiety, high anxiety/low avoidance). These findings collectively suggest that individuals with predominant avoidance features consistently manifest as minority subgroups across studies, with specific proportions potentially moderated by cultural contexts and mental health statuses (Hoenicka et al., 2022; Vaillancourt-Morel et al., 2022).

### 4.2 Relationships between attachment patterns and parenting behaviors

The low anxiety/avoidance profile represented the largest latent profile, closely matching secure attachment characteristics. Compared to other profiles, this group demonstrated more accepting behaviors and fewer rejecting behaviors in parenting. These findings align with Bowlby’s Internal Working Models theory—securely attached individuals develop positive self-other views, form stable emotional bonds, respond sensitively to children’s needs, and employ warm, supportive strategies (Bowlby, 1982; Kohlhoff et al., 2022; Yazdanimehr et al., 2023). After controlling for sex, attachment profiles did not differ significantly, and no significant differences in parenting behaviors emerged across sexes. These findings are consistent with previous studies (Dagan et al., 2021; Deneault et al., 2021; Vaillancourt-Morel et al., 2022), insecure attachment in either parent is associated with parent and child relationships, regardless of parental sex.

The study found that parents with high anxiety and moderate avoidance exhibited more rejecting behaviors than those with moderate anxiety/avoidance, particularly in terms of undifferentiated aggression. Parents with high anxiety and high avoidance showed more indifferent behaviors than those with moderate anxiety/avoidance. Put simply, compared to the two groups of parents with low-to-moderate anxiety characteristics, the two groups with high anxiety characteristics adopted more rejecting parenting behaviors. This further supports the disruptive role of insecure attachment in parenting (Dagan et al., 2021; Deneault et al., 2021). Attachment avoidance reflects a tendency to deactivate the attachment system, characterized by discomfort with intimacy and dependence in relationships, leading to negative parenting strategies (An and Kochanska, 2022; Nordahl et al., 2020). Attachment anxiety involves hyperactivating the attachment system, marked by strong fear of rejection and intense craving for closeness (Mikulincer and Shaver, 2007), also impairing responses to children’s needs. Notably, the high anxiety/high avoidance group scored higher (but without statistical significance) on three rejecting parenting dimensions than the high anxiety/moderate avoidance group. This suggests attachment anxiety may be more strongly linked to rejecting behaviors more strongly, which is consistent with studies on infants and mothers where maternal attachment anxiety mediated the intergenerational transmission of rejecting parenting (Kalfon Hakhmigari et al., 2021). Parents’ fear of rejection and excessive worry about closeness (e.g., fearing children’s rejection) may directly cause intrusive or distant parenting behaviors (Gross et al., 2023; Smith and South, 2020; Zhang et al., 2022). See Supplementary materials for analysis of attachment anxiety/avoidance links with parenting behaviors.

Rohner (2021) emphasized that parental acceptance (e.g., warmth, support) serves as a protective factor for children’s psychological adjustment, while rejection acts as a risk factor. Parental rejection not only increases risks of internalizing/externalizing problems (Chen and Volling, 2023; Rothenberg et al., 2022) but also elevates vulnerability to school bullying victimization (Chen et al., 2022). However, rejection in parenting does not equate to wholly negative behavior. Within warm parenting contexts, establishing boundaries and limits to guide children’s conduct benefits cognitive development and emotion regulation (Lanjekar et al., 2022; Morris et al., 2021). Recently, attachment and emotion focused parenting interventions (AE) have gained prominence as alternatives to behavioral parent training for children/adolescents. AE enhances parenting competence by helping parents understand and respond to children’s attachment/emotional needs, significantly reducing risks of internalizing/externalizing problems (Jugovac et al., 2022a,b). In summary, this study clarifies the concurrent association between parental attachment profiles and parenting behaviors, providing targeted insights for optimizing parent and child interactions and enhancing parental competence.

### 4.3 Limitations

First, reliance on self-report measures risks social desirability bias and common method variance. Second, regarding generalizability, several limitations must be noted. The sample was drawn from a single elementary school, which inherently limits the socioeconomic, regional, and cultural diversity of the participants. Consequently, the findings may not be fully representative of the broader population of parents of school-aged children. Specifically, participants were primarily urban parents with higher education and income levels compared to rural and low-income groups, further constraining broader applicability. Moreover, as a cross-sectional study (single time point), the design precludes causal inferences between parental attachment and parenting behaviors, as temporal relationships cannot be established. Third, the high avoidance/high anxiety profile had a small sample (*n* = 22, 3.5%). Bootstrap validation confirmed cluster stability, but low statistical power may affect reliability of parenting behavior conclusions for this profile, necessitating larger samples.

### 4.4 Recommendations

Despite these limitations, future work should verify these associations in diverse samples, including rural and lower income populations, to enhance generalizability. Additionally, longitudinal designs are needed to examine the temporal relationships and potential causal pathways between parental attachment profiles and parenting behaviors. Further research should also explore how specific parenting behaviors mediate or moderate the links between parental attachment and child mental health outcomes.

## 5 Conclusion

This study used LPA to identify four heterogeneous parental attachment profiles among parents of school-age children: low anxiety/avoidance, high anxiety/moderate avoidance, moderate anxiety/avoidance, and high anxiety/avoidance. Consistent with previous research, findings revealed significant associations between parental attachment patterns and parenting behaviors. Parents with low anxiety/avoidance demonstrated substantially higher levels of accepting parenting behaviors and significantly lower levels of rejecting parenting behaviors than other profiles. Additionally, parents exhibiting high avoidance or anxiety traits employed more rejecting parenting behaviors regardless of sex. Collectively, these results underscore the critical association between parental attachment patterns and parenting decisions, providing an empirical basis and insights for enhancing parental competence.

## Data Availability

The raw data supporting the conclusions of this article will be made available by the authors upon reasonable request.
