# Supplementary material for: Latent profiles of parental attachment styles and their associations with parenting behaviors among parents of school-aged children
Source: Front Psychol. 2026 May 4;17:1691655. doi: 10.3389/fpsyg.2026.1691655 (PMC13180531; doi:10.3389/fpsyg.2026.1691655)
Supplement: Supplementary file 1 [file Table_1.docx]

**Contents**

[1.Supplementary Methods 1](#_Toc206414833)

[1.1Procedure 1](#_Toc206414834)

[1.2Statistical analysis 1](#_Toc206414835)

[2.Supplementary Results 2](#_Toc206414836)

[2.1 Mediation analysis: Acceptance as the dependent variable 2](#_Toc206414837)

[2.2 Mediation analysis: ​Rejection as the dependent variable 2](#_Toc206414838)

[Supplementary Reference: 3](#_Toc206414839)

# 1.Supplementary Methods

## 1.1Procedure

## Consistent with significant intercorrelations among attachment anxiety, attachment avoidance, and parental acceptance-rejection behaviors identified in this study, we conducted four mediation analyses to systematically examine distinct mechanistic roles of anxiety versus avoidance in predicting specific parenting behavior patterns. The models specified: (1) Anxiety as independent variable → Avoidance as mediator → Acceptance as dependent variable (2) Avoidance → Anxiety → Acceptance (3) Anxiety → Avoidance → Rejection (4) Avoidance → Anxiety → Rejection

## 1.2Statistical analysis

Statistical analyses were performed using SPSS Statistics 27.0. Mediation effects were tested via Hayes' PROCESS macro (v4.0) [1] with 95% bias-corrected bootstrapped confidence intervals based on 5,000 resamples to examine relationships between attachment patterns and parenting behaviors.

# 2.Supplementary Results

## Mediation analysis: Acceptance as the dependent variable

Regression analyses supported a significant mediation model with attachment anxiety as the independent variable, avoidance as mediator, and parental acceptance as outcome (Tables 1/2). Step 1 revealed attachment anxiety significantly predicted avoidance (β = 0.253, p < 0.001). In Step 2, both anxiety (β = -0.125, p < 0.001) and avoidance (β = -0.131, p = 0.030) significantly predicted parental acceptance. ​Bootstrap analyses​ confirmed a significant standardized indirect effect (β = -0.033, 95% CI [-0.052, -0.015]), indicating attachment avoidance partially mediates the relationship between attachment anxiety and accepting parenting behaviors.

Conversely, the alternative model positioning attachment avoidance as the independent variable and attachment anxiety as the mediator ​did not demonstrate significant mediation effects​ in predicting parental acceptance.

Table 1. Regression Results for Mediation Analysis (N = 416)​​

| Predictor | β | SE | t | p | 95% CI |
| --- | --- | --- | --- | --- | --- |
| Step 1​ Dependent Variable: Avoidance | | | | | |
| Anxiety | 0.253 | 0.024 | 10.437 | <0.001 | [0.206, 0.301] |
| Step 2​ Dependent Variable: Acceptance | | | | | |
| Anxiety | -0.125 | 0.033 | -3.763 | <0.001 | [-0.191, -0.060] |
| Avoidance | -0.131 | 0.060 | -2.182 | 0.030 | [-0.249, -0.013] |

Note: β = unstandardized coefficients; CI = confidence interval

Table 2. Mediation Effect Decomposition​

| Effect Type | Value | Boot SE | Boot 95% CI | Relative Size |
| --- | --- | --- | --- | --- |
| Total Effect | -0.159 | - | - | 100% |
| Direct Effect | -0.125 | 0.033 | [-0.191, -0.060] | 79.1% |
| Indirect Effect | -0.033 | 0.015 | [-0.065, -0.005] | 20.9% |

Note: Bootstrap resampling = 5000 times; significant indirect effect confirmed by CI excluding 0

## 2.2 Mediation analysis: ​Rejection as the dependent variable

A significant mediation model emerged with avoidance as independent variable, anxiety as mediator, and rejection as outcome (Tables 3/4). Step 1 demonstrated avoidance significantly predicted anxiety (β = 0.823, p < 0.001). In Step 2, anxiety significantly predicted rejecting parenting behaviors (β = 0.227, p < 0.001), while avoidance’s direct effect on rejection was non-significant (β = 0.071, p = 0.205). ​Bootstrap analyses​ revealed a significant standardized indirect effect (β = 0.187, 95% CI [0.142, 0.233]), indicating attachment anxiety ​fully mediates​ the relationship between attachment avoidance and rejecting parenting behaviors.

Conversely, the alternative model specifying attachment anxiety as the independent variable and attachment avoidance as the mediator ​showed no significant mediation effects​ in predicting parental rejection.

Table 3. Regression Results for Mediation Analysis (N = 416)

| Predictor | β | SE | t | p | 95% CI |
| --- | --- | --- | --- | --- | --- |
| Step 1​ Dependent Variable: Anxiety | | | | | |
| Avoidance | 0.823 | 0.079 | 10.437 | <0.001 | [0.668, 0.978] |
| Step 2​ Dependent Variable: Rejection | | | | | |
| Avoidance | 0.071 | 0.056 | 1.270 | 0.205 | [-0.039,0 .182] |
| Anxiety | 0.227 | 0.031 | 7.291 | <0.001 | [0.166, 0.288] |

Note: β = unstandardized coefficients; CI = confidence interval;

​Table 4. Mediation Effect Decomposition​

| Effect Type | Value | Boot SE | Boot 95% CI | Relative Size |
| --- | --- | --- | --- | --- |
| Total Effect | 0.258 | - | - | 100% |
| Direct Effect | 0.071 | 0.056 | [-0.039, 0.182] | 27.6% |
| Indirect Effect | 0.187 | 0.033 | [0.128, 0.256] | 72.4% |

Note: Bootstrap resamples = 5,000; CI for indirect effect does not contain 0 (significant)

​

# Supplementary Reference:

1. Hayes, A.F., *Introduction to mediation, moderation, and conditional process analysis: A regression-based approach*. Introduction to mediation, moderation, and conditional process analysis: A regression-based approach. 2013, New York, NY, US: The Guilford Press. xvii, 507-xvii, 507.
