# Supplementary material for: Latent profiles of parental attachment styles and their associations with parenting behaviors among parents of school-aged children
Source: Front Psychol. 2026 May 4;17:1691655. doi: 10.3389/fpsyg.2026.1691655 (PMC13180531; doi:10.3389/fpsyg.2026.1691655)
Supplement: Supplementary file 2 [file Table_2.docx]

**Contents**

# 1.Procedure

All statistical analyses were performed using Mplus 8.3. Latent profile analysis (LPA) was conducted to identify parental attachment profiles based on the 18-item Adult Attachment Scale (AAS). After selecting the 3-profile solution, the Bolck-Croon-Hagenaars (BCH) method [30] was used to examine differences in parenting behaviors across profiles, accounting for classification uncertainty to ensure robust comparisons.

# 2.Supplementary Results

**Table 1 Group differences in attachment among three latent profiles.**

|  | Class 1(n=208) | Class 2(n=184) | Class 3(n=24) | Significant differences |
| --- | --- | --- | --- | --- |
| Avoidance | 2.47 ± 0.37 | 2.90 ± 0.37 | 3.12 ± 0.37 | 3＞2＞1*** |
| Anxiety | 1.45 ± 0.43 | 2.49 ± 0.43 | 3.72 ± 0.43 | 3＞2＞1*** |

Note: n= number; values are presented as Mean ± Standard Deviation; ****p* < 0.001

Latent profile analysis identified three distinct attachment profiles. Profile 1 (n = 208), the largest subgroup, showed the lowest levels of attachment avoidance and anxiety, reflecting a secure attachment pattern. Profile 2 (n = 184) was characterized by moderate avoidance and anxiety, representing a moderately insecure attachment pattern. Profile 3 (n = 24), the smallest subgroup, exhibited the highest levels of both attachment anxiety and avoidance, indicating a highly anxious and avoidant attachment pattern. All pairwise comparisons were significant (*p* < 0.001), revealing a clear gradient of increasing attachment insecurity from Profile 1 to Profile 3.

**Table 2 Equality tests of means across attachment profiles for acceptance/rejection (BCH).**

|  | Class 1 | Class 2 | Class 3 | Chi-square | Significant differences(*p*) |
| --- | --- | --- | --- | --- | --- |
| Acceptance | 3.269 | 3.040 | 2.787 | 22.606 | 1 vs 2 (<0.001);  1 vs 3 (0.002);  2 vs 3 (0.123) |
| Rejection | 1.336 | 1.673 | 2.062 | 56.697 | 1 vs 2 (<0.001)  1 vs 3 (<0.001)  2 vs 3 (0.017) |
| Hostility | 8.301 | 10.536 | 12.675 | 46.697 | 1 vs 2 (<0.001)  1 vs 3 (<0.001);  2 vs 3 (0.038) |
| Coldness | 8.070 | 10.189 | 12.732 | 63.652 | 1 vs 2 (<0.001)  1 vs 3 (<0.001)  2 vs 3 (0.009) |
| Undifferentiated Rejection | 5.000 | 6.049 | 7.591 | 28.739 | 1 vs 2 (<0.001)  1 vs 3 (<0.001)  2 vs 3 (0.038) |

Note: BCH = Bolck-Croon-Hagenaars method;

The BCH procedure was used to examine differences in acceptance/rejection related parenting behaviors among the three latent profiles of attachment (Table 2). Bonferroni correction was applied to adjust the significance level (adjusted α = 0.05/3 ≈ 0.0167). The results indicated that Class 1 (the secure attachment profile) demonstrated significantly higher levels of acceptance than the other two latent profiles (*χ²* = 22.606, *df* = 2, *p* < 0.001). Compared to Class 1 (low attachment insecurity) and Class 2 (moderate attachment insecurity), Class 3 (high attachment insecurity) exhibited higher levels of rejection, hostility, coldness, and undifferentiated rejection. Specifically, Class 1 scored significantly higher than Class 2 in acceptance (*χ²* = 13.955, *df* = 2, *p* < 0.001), and Class 1 scored significantly higher than Class 3 in acceptance (*χ²* = 9.663, *df* = 2, *p* = 0.002). Class 3 scored significantly higher than Class 2 in coldness (*χ²* = 6.846, *df* = 2, *p* = 0.009). Class 3 exhibited significantly higher undifferentiated rejection compared to Class 1 (*χ²* = 13.982, *df* = 2, *p* < 0.001) and Class 2 (*χ²* = 4.288, *df* = 2, *p* = 0.038, not significant after Bonferroni correction). Despite differing in attachment avoidance and anxiety levels, Class 2 and Class 3 showed no significant differences in acceptance (*χ²* = 2.378, *df* = 2, *p* = 0.123), hostility (*χ²* = 4.311, *df* = 2, *p* = 0.038), and undifferentiated rejection (*χ²* = 4.288, *df* = 2, *p* = 0.038) after Bonferroni correction-
