# Supplementary material for: Latent profiles of parental attachment styles and their associations with parenting behaviors among parents of school-aged children
Source: Front Psychol. 2026 May 4;17:1691655. doi: 10.3389/fpsyg.2026.1691655 (PMC13180531; doi:10.3389/fpsyg.2026.1691655)
Supplement: Supplementary file 3 [file Table_3.docx]

Supplementary Direct Entry Regression Analysis

This study utilized SPSS version 27 to conduct multiple regression analysis using the standard enter method. Age, gender (male [1], female [2]), and scores on attachment avoidance and anxiety dimensions were included as predictor variables to examine their predictive effects on parental rearing styles (acceptance/rejection).

For the rejection parenting style, the regression model showed that the four predictor variables together explained 16.4% of the variance in rejection parenting style (*F* (4, 411) = 20.099, *p* < 0.001), indicating a moderate overall explanatory power. Among the predictors, gender (*β* = -0.02, *p* = 0.655), age (*β* = -0.02, *p* = 0.655), and attachment avoidance (*β* = -0.02, *p* = 0.655) did not have statistically significant predictive effects on rejection parenting style. In contrast, attachment anxiety had a significant positive predictive effect on rejection parenting style (*β* = 0.365, *p* < 0.001).

For the acceptance parenting style, the regression model revealed that the four predictor variables jointly explained 7.7% of the variance in acceptance parenting style (*F* (4, 411) = 8.610, p < 0.001). Among them, gender (*β* = 0.047, *p* = 0.331) and age (*β* = -0.014, *p* = 0.771) did not show statistically significant predictive effects on acceptance parenting style. Attachment avoidance (*β* = -0.119, p = 0.027) and attachment anxiety (*β* = -0.200, *p* < 0.001) both had significant predictive effects on acceptance parenting style.
